# Deletion of Cdc42 in embryonic cardiomyocytes results in right ventricle hypoplasia

**DOI:** 10.1186/s40169-017-0171-4

**Published:** 2017-11-03

**Authors:** Yang Liu, Jian Wang, Jieli Li, Rui Wang, Binu Tharakan, Shenyuan L. Zhang, Carl W. Tong, Xu Peng

**Affiliations:** 1 Department of Medical Physiology, College of Medicine, Texas A&M University, Temple, USA; 2 Department of Obstetrics and Gynecology, Baylor Scott & White Health, Temple, USA; 3 Yangpu District Central Hospital, Tongji University, Shanghai, China; 4 Department of Surgery, Baylor Scott & White Health, Temple, USA; 5 Internal Medicine/Cardiology Division, Baylor Scott & White Health, Temple, USA

**Keywords:** GTPase, Cdc42, Heart development, Right ventricle development

## Abstract

**Background:**

Cdc42 is a member of the Rho GTPase family and functions as a molecular switch in regulating cytoskeleton remodeling and cell polarity establishment. Inactivating Cdc42 in cardiomyocytes resulted in embryonic lethality with heart developmental defects, including ventricular septum defects and thin ventricle wall syndrome.

**Findings:**

In this study, we have generated a Cdc42 cardiomyocyte knockout mouse line by crossing Cdc42/flox mice with myosin light chain 2a (MLC2a)-Cre mice. We found that the deletion of Cdc42 in embryonic cardiomyocytes resulted in an underdeveloped right ventricle. Microarray analysis and real-time PCR data analysis displayed that the deletion of Cdc42 decreased dHand expression level. In addition, we found evaginations in the ventricle walls of Cdc42 knockout hearts.

**Conclusion:**

We concluded that Cdc42 plays an essential role in right ventricle growth.

## Introduction

The mammalian heart is the first functional organ formed during embryogenesis, and it uninterruptedly pumps blood throughout adulthood. Interfering with the process of cardiac development results in congenital heart diseases, which affect 40,000 newborns per year in the USA [1–3]. Cardiac morphogenesis and maturation require the deployment of multiple cell lineages that are derived from the primary heart field, secondary heart field, and neural crest cells [4–6]. Initially, the cardiac progenitors that derive from the primary heart field migrate toward the midline of the embryo to fuse and form the primary heart tube. Subsequently, the primary heart tube is elongated by recruiting cells that are generated from the second heart field. Finally, the heart tube undergoes rightward looping and chamber formation [5, 6].

The cardiac progenitors of the right and left ventricles come from different sources [7]. The left ventricle cardiac progenitors are mainly derived from the primary heart field, and the right ventricle cardiomyocytes are from the secondary heart field [8]. Development of the right and left ventricles is regulated by distinct developmental programs. Interfering ventricle-specific transcription factors and/or their effectors can cause sole ventricle developmental defects [9]. Hypoplastic right heart syndrome is a rare congenital heart disease that is characterized by an underdeveloped right ventricle [10, 11]. However, the molecular mechanisms that regulate right ventricle development remain elusive. Three papers published in 2001 reported that cardiac progenitors deriving from the secondary heart field contributed to heart tube elongation and right ventricle development in chicks and mice [12–14]. Interfering with cardiac progenitor recruitment from the secondary heart field resulted in a small right ventricle. Insulin gene enhancer protein-1 (Isl-1) is expressed in the entire secondary heart field and then in the bilateral pharyngeal mesoderm. The inactivation of Isl-1 prevented formation of the right ventricle and outflow track [15]. Right ventricle development is dependent on ventricle-specific transcriptional factors, including myocyte enhancer factor 2c (MEF2c) and heart- and neural crest derivatives-expressed protein 2 (dHand), as well as the recruitment of cardiac progenitors from the secondary heart field [16]. MEF2c is required for right ventricle formation, and its promoter region contains Isl-1 and GATA binding sites [17]. dHand is a home box transcription factor that is predominantly expressed in the right ventricle [18]. dHand cannot be directly regulated by Isl-1, but can be regulated by BOP, a downstream effector of MEF2c. Inactivation of dHAND resulted in right ventricle hypoplasia. Furthermore, fibroblast growth factor 10 (FGF10) as well as bone morphogenetic protein (BMP)-initiated signal transduction are involved in right ventricle development [6]. However, the role of signal transduction in regulating right ventricle development remains elusive.

Cdc42 is a Ras GTPase superfamily member that is essential for regulating establishment of actin polymerization [19]. In response to extracellular stimulation (growth factors, shear stress, etc.), Cdc42’s activity is tightly controlled by GTPase-activating proteins (GAPs), guanine nucleotide exchange factors (GEF) and guanine nucleotide dissociation inhibitors (GDI) [19, 20]. Recently, we and others have reported that Cdc42 plays an important role in heart development in both mice and drosophila [21, 22]. In addition, the deletion of Cdc42 resulted in small right ventricles and enlarged right atria in the adult mice [22]. To further investigate the role of Cdc42 in heart development, we examined the role of Cdc42 in right ventricle development and found that Cdc42 is required for right ventricle growth.

## Materials and methods

### Generation of Cdc42 cardiomyocyte knockout mice

Cdc42 cardiomyocyte knockout mice were generated by crossing Cdc42/flox mice with MLC-2a Cre mice, as previously reported [22]. The protocol of animal studies was reviewed and approved by the Animal Care and Use Committee of Texas A&M Health Science Center.

### Histology analysis

E14.5 embryos were harvested and fixed with 4% paraformaldehyde. After dehydration, the embryos were embedded in paraffin and sectioned in 4 micrometers. Hematoxylin and eosin (H&E) staining was then performed.

### Microarray and real-time PCR analysis

E14.5 embryonic hearts were harvested and RNA was isolated with the Qiagen RNeasy mini kit (QIAGEN, CA). cRNA was synthesized using standard protocols and then applied to an Affymetrix Gene Chip Mouse Exon 1.0 ST Array (Affymetrix, CA). cDNA was synthesized with SuperScript® III Reverse Transcriptase (Invitrogen, CA). Real-time PCR analysis was performed as previously reported [22]. We harvested six control and knockout embryonic hearts for real-time PCR analysis.

### Statistical analysis

Student’s T test was used to determine statistical significance. P values < 0.05 were considered significant. Values for all measurements were expressed as mean ± standard deviation.

## Results

**Fig. 1:**
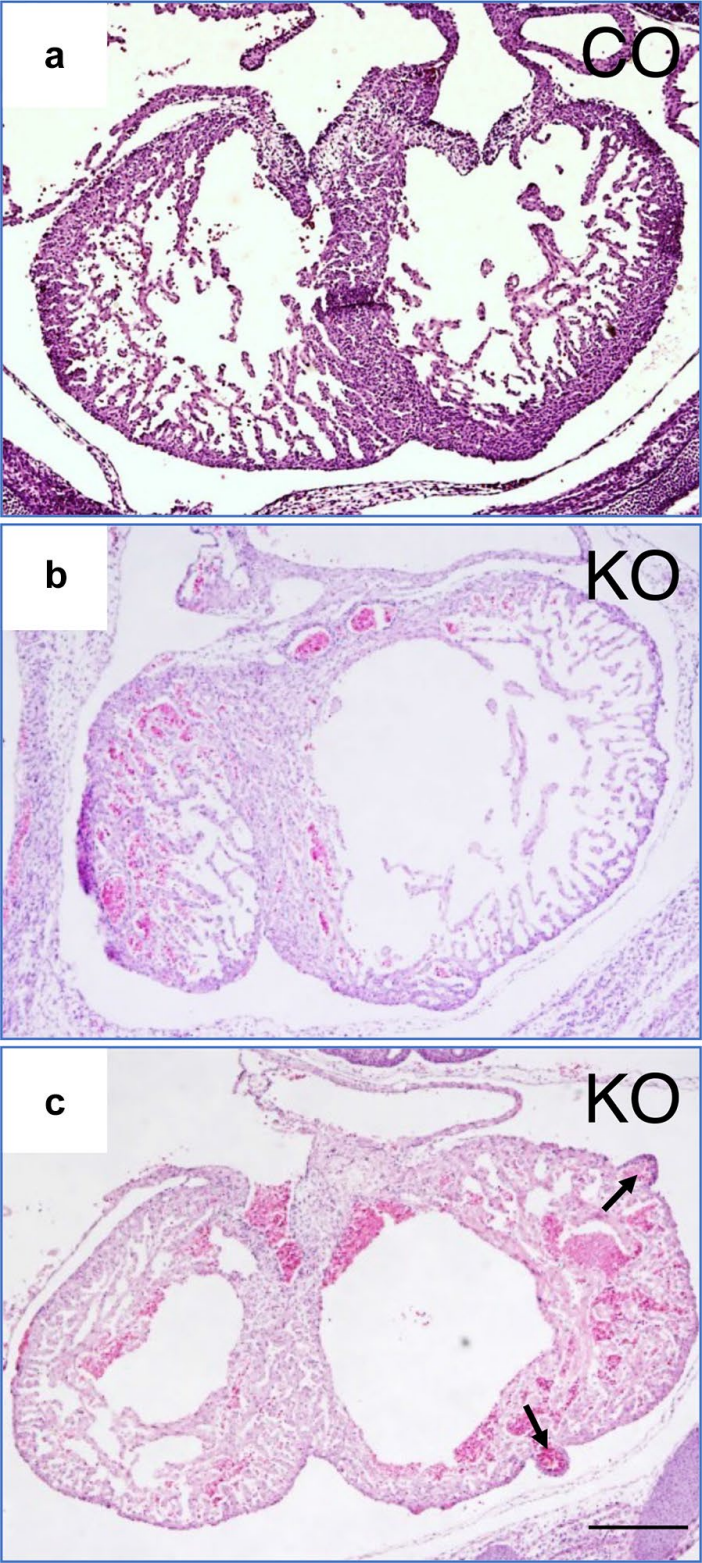
Inactivation of Cdc42 in embryonic cardiomyocytes impaired right ventricle growth. Histological transverse sections through the hearts from E14.5 control (**a**) and knockout embryos (**b**, **c**). H&E staining showed a smaller right ventricle in the different section levels of the knockout (**b**, **c**). Black arrows indicate evaginations in the ventricle wall. Scale bar: 250 μm

To determine the role of Cdc42 in heart development, we created a mouse line by crossing Cdc42/flox mice with MLC2a-Cre mice, which inactivated Cdc42 in the embryonic cardiomyocytes [22]. As reported before, we found that deleting Cdc42 in embryonic cardiomyocytes caused lethality with heart development defects (thin ventricle walls). Interestingly, we noted that the deletion of Cdc42 impaired right ventricle development. In the control, the size of the right ventricle was comparable to the left ventricle at E14.5 (Fig. 1a). On the other hand, we found the size of right ventricle to be smaller than that of the left in Cdc42 knockout embryo serial sections. In four out of seven examined knockout embryos, the size ratio between right ventricle and left ventricle is closed to 60% in knockout and 100% in the control (Fig. 1b), indicating that Cdc42 is required for right ventricle growth. Moreover, we noted abnormal evaginations in the left ventricle wall of the knockout heart (Fig. 1c).

**Fig. 2:**
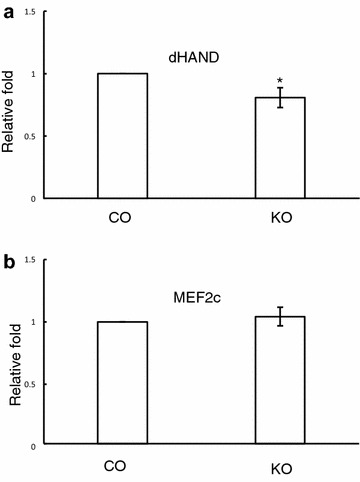
Microarray analysis of dHand and MEF-2c expression levels in E14.5 embryonic hearts. **a** The expression level of dHand was down-regulated in the knockout hearts. **b** MEF-2c expression levels were comparable between control and knockout. *P < 0.05

**Fig. 3:**
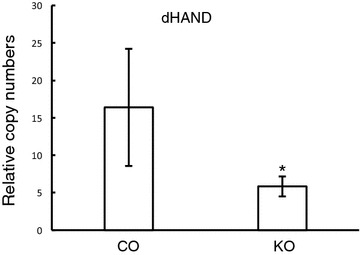
Real-time PCR analysis of dHand expression levels. The expression level of dHand was significantly decreased in Cdc42 knockout heart. *P < 0.05

Microarray analysis was performed to investigate the mechanisms of Cdc42 in heart development. RNA from E14.5 embryonic hearts was isolated and then blotted with microarray slides. We checked the transcription factors involved in right ventricle development and found that dHand expression levels were significantly decreased in the knockout compared to the control (Fig. 2a). MEF2c expression levels were comparable between control and knockout (Fig. 2b). To further confirm the microarray data, we performed real-time PCR analysis. Our results showed that the deletion of Cdc42 down-regulated dHand, compared to the control (Fig. 3a). This indicated that Cdc42 may be involved in regulating right ventricle development through dHand during embryogenesis.

## Discussion

Congenital heart disease is the most common birth defect in the world and can be induced by genetic mutations and/or exposures to environmental risk factors [23]. In our previous studies, we have reported that deleting Cdc42 in cardiomyocytes resulted in thin ventricle walls and ventricular septum defects. In addition, we also observed dilated right atria and small right ventricles in Cdc42 knockout mice [22]. In the current study, we analyzed Cdc42 knockout embryonic hearts using histology analysis and found that the deletion of Cdc42 resulted in small right ventricles. Additionally, the deletion of Cdc42 in the embryonic hearts decreased dHand expression.

One of the most dramatic phenotypes of the Cdc42 knockout mice were the underdeveloped right ventricles. The size difference between the left and right ventricles cannot be explained by cardiomyocyte proliferation defects because the deletion of Cdc42 affected cardiomyocyte proliferation in both left and right ventricles. In addition, it was documented that many other genes, including focal adhesion kinase, etc., are important for cardiomyocyte proliferation. However, inactivation of those genes did not affect the right ventricle size [24]. Therefore, the ventricle size and ventricle cardiomyocyte proliferation may be controlled by independent regulatory mechanisms. In our case, we inactivated Cdc42 in cardiomyocytes using MLC2a-Cre. Thus, it was impossible to affect cardiac progenitor recruitment from the secondary heart field because MLC2a was expressed in differentiated cardiomyocytes instead of in cardiac progenitors of the secondary heart field.

It was documented that the regulatory transcription factors in the left and right ventricles are different [25]. For example, dHand is specifically expressed in the right ventricle and is required for right ventricle and cardiac outflow formation [18]. In contrast, eHand is expressed in the left ventricle and inactivation of eHand impaired left ventricle growth and maturation [26]. In our study, we found that the inactivation of Cdc42 impaired dHand expression and resulted in a small right ventricle. Therefore, it is possible that Cdc42 controls right ventricle growth through dHand. However, it remains unclear how Cdc42 regulates dHand expression during heart development.

FGF10 was detected only in the right ventricle during embryonic heart development [12]. Consistently, the inactivation of FGF10 resulted in an underdeveloped right ventricle. It was reported that FGF stimulation induced BNIP-2 (a member of the BCL2/adenovirus E1B 19 kd-interacting protein) phosphorylation. The phosphorylation of BNIP-2 decreased its binding and GAP-like activity toward Cdc42, keeping Cdc42 in its active status [27]. Therefore, it is possible that Cdc42 is involved in FGF10-initiated signal transduction and that the inactivation of Cdc42 impaired FGF10-induced right ventricle formation.

In addition to the smaller right ventricles found in Cdc42 knockout mice, we also noted that the inactivation of Cdc42 resulted in ventricle wall evagination. Previously, we reported that Cdc42 is required for maintaining cardiomyocyte cell–cell adhesion and that the inactivation of Cdc42 impaired cardiomyocyte-adherent junction formation [22]. It was reported that the activated Cdc42 can prevent IQGAP1 from binding to β-catenin, weakening the Cadherin-Catenin-actin-based cell–cell adherent junctions [28]. Therefore, it is possible that the inactivation of Cdc42 increased the binding between β-catenin and IQGAP1 and disrupted *N*-cadherin–catenin–actin-based cell–cell adherent junctions. Because of the increased pressure inside the ventricle during heart development, the weakest section of the ventricle wall may balloon out to form abnormal evaginations.
